# Supplementary material for: Photocatalytic conversion of sugars to 5-hydroxymethylfurfural using aluminium(III) and fulvic acid
Source: Nat Commun. 2023 Aug 1;14:4609. doi: 10.1038/s41467-023-40090-7 (PMC10393994; doi:10.1038/s41467-023-40090-7)
Supplement: Supplementary file 1 — Supplementary Information [file 41467_2023_40090_MOESM1_ESM.pdf]

Supplementary Information for

Photocatalytic conversion of sugars to 5-hydroxymethylfurfural using aluminium(III) and fulvic acid

Tana Tana[1,2], Pengfei Han[2,3,*], Aidan J. Brock[2], Xin Mao[2], Sarina Sarina[2], Eric R. Waclawik[2], Aijun Du[2], Steven E. Bottle[2], Huai-Yong Zhu[2]*

[1] School of Mongolian Medicine, Inner Mongolia Minzu University, Tongliao, Inner Mongolia 028000, China
[2] School of Chemistry and Physics, Queensland University of Technology, Brisbane QLD4001, Australia
[3] College of Chemistry and Chemical Engineering, Hunan University Changsha, 410082, China

Correspondence: pengfeihan@hnu.edu.cn (PH.), hy.zhu@qut.edu.au (H.Z.)

TABLE OF CONTENT:

Section 1: Anion effects on catalytic activity 4

Section 2: Influence of atmosphere on sugar transformation to HMF 5

Section 3: Observations on the non-illuminated thermal reaction......................... 6

Section 4: Evidence of complexation between Al^{3+} and FA 7

Section 5: The experiment under sunlight.. 11

Section 6: Photoluminescence emission spectra of the catalytic system and its separate components.. 12

Section 7: UV-vis spectra of FA-polyphenol-glucose complexes after irradiation under different wavelengths.. 15

Section 8: NMR studies on the interaction of FA with Al^{3+} 16

Section 9: Calculated frontier molecular orbitals for aluminium(III)-pyrogallol-glucose complexes.. 21

Section 10: GPC analysis of FA 22

Section 11: Solvent effect... 23

Section 12: Carbon balance determination 24

References.. 24

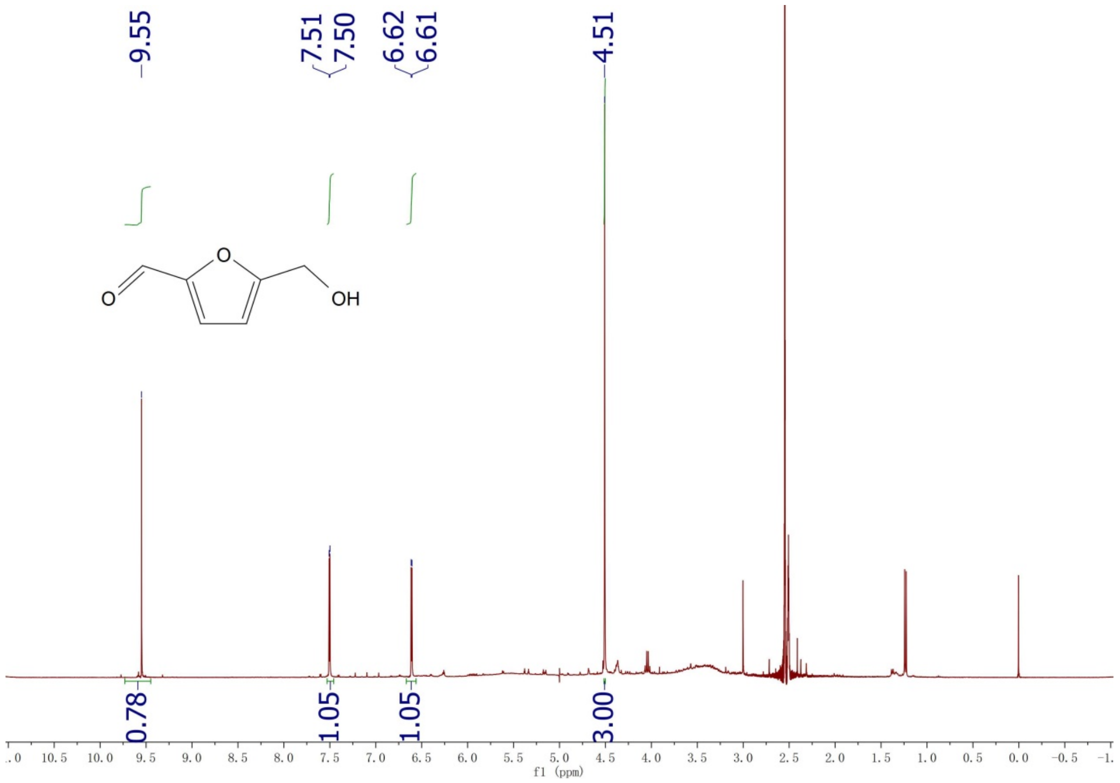

Supplementary Fig. 1. NMR spectrum of 5-HMF produced from D-glucose. ^{1}H NMR (400 MHz, DMSO-*d6*) δ 9.55 (s, 1H), 7.51 (d, *J*=4.0 Hz, 1H), 6.61 (d, *J*=4.0 Hz, 1H), 4.51 (s, 3H).

Section 1: Anion effects on catalytic activity

Conducting the reaction with aluminium acetate or aluminium sulfate showed no conversion of D-glucose to HMF. This highlights the critical need for appropriate control of ligand selection for this reaction. The anionic acetate ligands coordinate strongly to the hard Lewis acid aluminium(III), forming small, stable oxo-bridged clusters[1], in which the acetate ligands will be difficult to displace by other ligands and substrate molecules, thereby preventing the formation of the active catalytic species. Aluminium sulfate has a poor solubility in DMSO, and thus cannot form active photocatalytic species. In contrast, the use of aluminium chloride afforded high yields (50%). This salt is more soluble in DMSO than the sulfate and does not form highly chelated clusters like acetate. This result suggests that the Cl^- anion does not play a significant role in the active catalytic species.

Section 2: Influence of atmosphere on sugar transformation to HMF

Our photocatalytic system is indeed affected by oxygen, performing significantly better under an inert atmosphere (Table 1). This can be explained by oxidation of the catechol ligand to the semiquinone and subsequent quinone forms under an oxygen atmosphere, changing the electronic properties of the catalyst to a catalytically inert form. Indeed, while attempting to isolate aluminium catecholate species in solution under aerobic conditions, a dark green colour typical of aluminium semiquinonate complexes[2] was observed (proved by LC-MS results shown in Supplementary Fig. 2). The incorporation of additional protections against ligand oxidation may provide a route to the minimisation of catalyst deactivation, increasing the efficiency of this photocatalytic system.

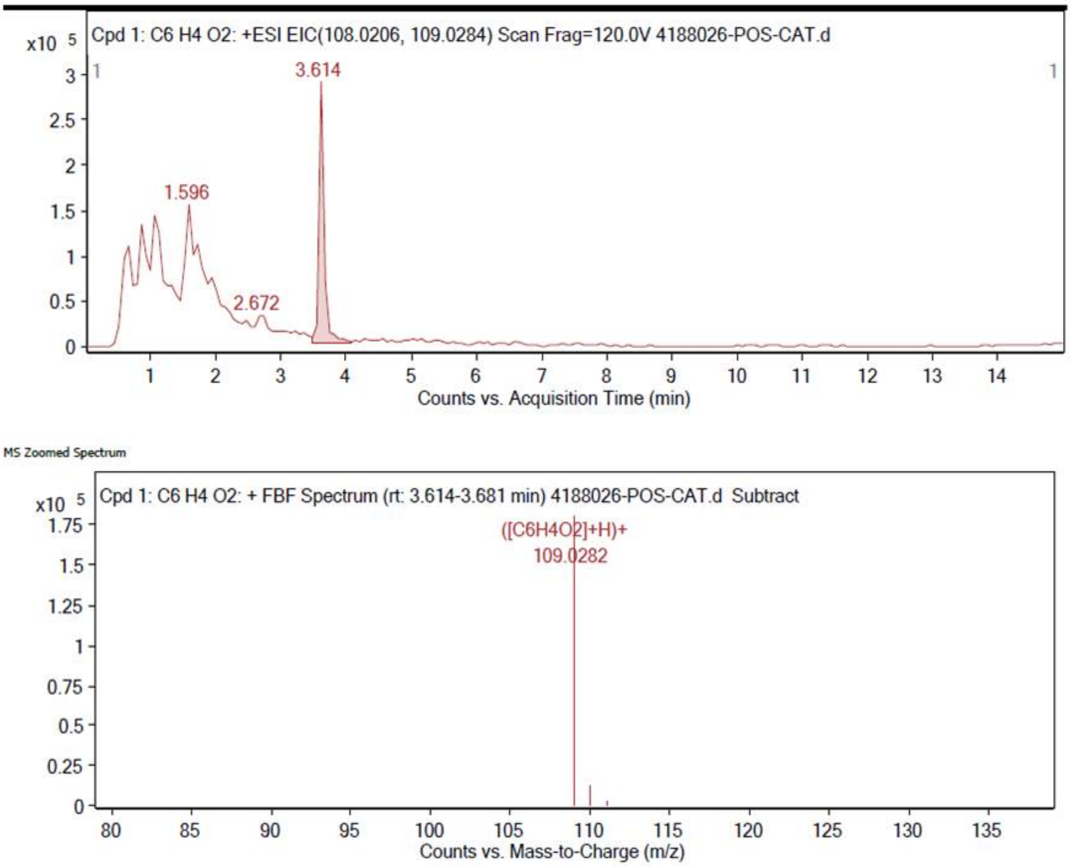

Supplementary Fig. 2. Detection of the product obtained in the reaction under aerobic conditions refers to Section 2 by LC-MS (Agilent 1290 uplc- Agilent qtof 6550). Reaction conditions: 0.01 M of aluminium salt, 8.0 g L^{-1} catechol, 0.1 M glucose/DMSO, 1.2 W cm^{-2} of light intensity (400-800 nm wavelength), 80 ^{o}C, 20 h, 1 atm air atmosphere. m/z (ESI) calculated for (C6H4O2)$[M+H]^+$:109.02, found: 109.02.

Section 3: Observations on the non-illuminated thermal reaction

HMF yield of the reactions in the dark increased moderately when the reaction temperature was increased from 80 ^{o}C to 120 ^{o}C, while further increasing temperature to 130 ^{o}C results in higher HMF yields (15-17%). The thermal reaction conducted at 140 ^{o}C achieved the maximum HMF yield of 38%, and the yield decreased to approximately 20% at 170 ^{o}C. The results are comparable with the reported HMF yields obtained using only aluminium salts as the catalyst over a similar reaction temperature range.[3-7] The presence of FA has a negligible contribution to HMF yield in the dark reaction. The color of the final reaction mixture changed from light yellow (the colour of FA in DMSO) to dark brown when the reaction temperature increased to 170 ^{o}C. The main reason is likely the formation of humin, a by-product that often occurs during sugar conversion to HMF.[8] The decreased HMF yield at temperatures over 150 ^{o}C may be due to large amounts of humin formation by condensation of HMF and sugars.

We found that 80 ^{o}C is the optimal reaction temperature on photocatalytic sugar transformation to HMF. This temperature is achievable by solar water heater. Thus, sunlight can provide most of the energy that the reaction needed.

Supplementary Table 1. Influence of reaction temperature on sugar transformation to HMF.

Entry	Temperature (°C)	Product photos	Yield (%)
1	80	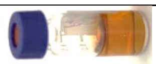	59
2	90	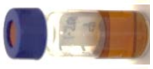	53.1
3	100	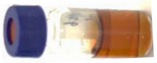	29.5

Reaction conditions: 0.01 mmol of aluminium salt, 8 mg of fulvic acid, 18 mg of D-glucose, 1 mL of DMSO as the solvent, 1.2 W cm^{-2} of light intensity (400-800 nm wavelength), 20 h of reaction time, 1 atm argon atmosphere. The samples were diluted 10 times with methanol and filtered before GC measurements.

Section 4: Evidence of complexation between Al^{3+} and FA

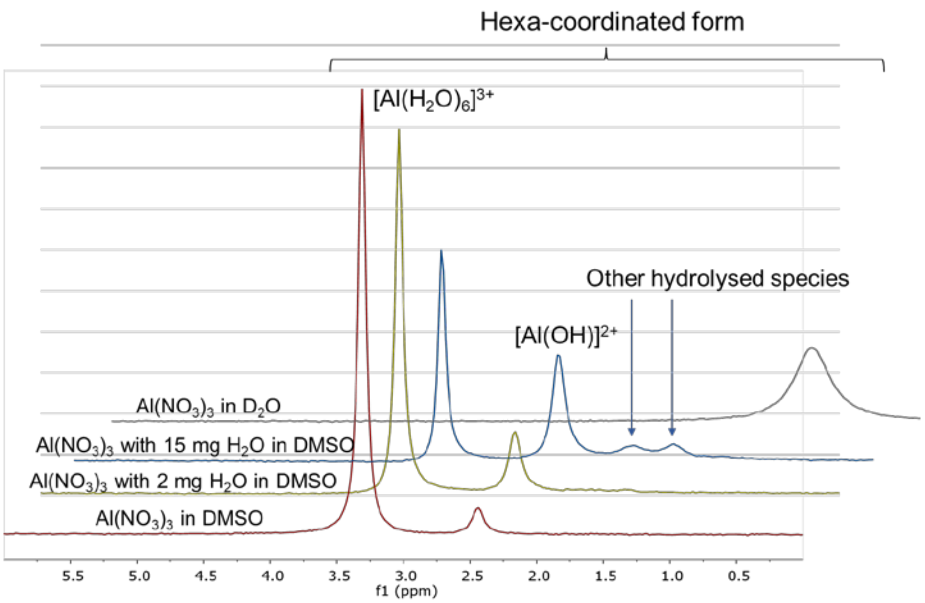

Supplementary Fig. 3. ^{27}Al NMR spectra of 8 mM $Al(NO_3)_3$ in pure DMSO-*d6* (red), and with adding 2 mg H_2O (green), 15 mg H_2O (blue) and $Al(NO_3)_3$ in pure D_2O (grey).

As the crystalline hydrate, $Al(NO_3)_3{\cdot}9H_2O$ used in this study has a structure with six of the water molecules residing in the first hydration shell of the Al^{3+} ions, the highest peak at δ = 3.3 ppm should correspond to the dominant species $[Al(H_2O)_6]^{3+}$. To determine the peak assignments of δ = 2.4 ppm, a small amount of water was added to the solution. The results show that with the increasing amount of water, the peak at δ =3.3 ppm decreases while the peak at δ =2.4 ppm increases significantly.

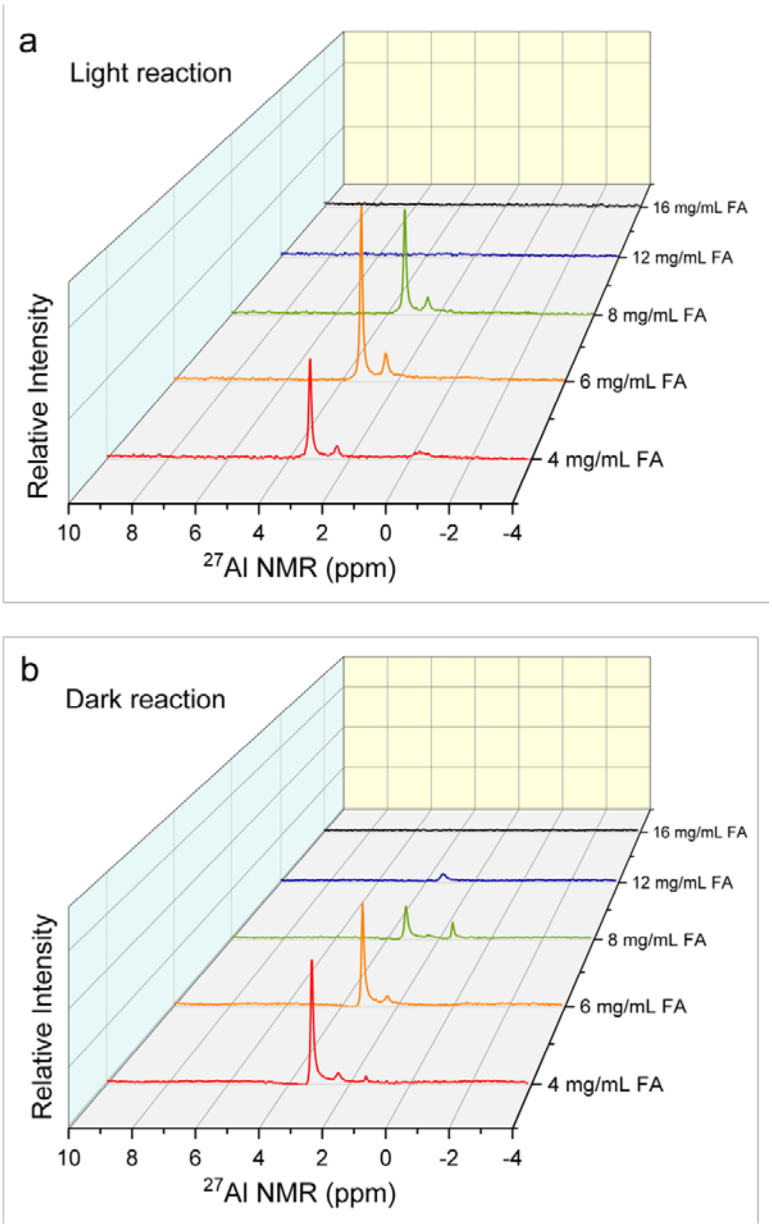

Supplementary Fig. 4. ^{27}Al NMR spectra of reaction mixtures obtained from glucose conversion to HMF using Al^{3+} and different FA concentrations after 20 h visible light irradiation (a) and in the dark (b). Reaction conditions were the same as Fig. 1e.

Supplementary Fig. 4 shows ^{27}Al NMR analysis of reaction mixtures obtained from glucose conversion to HMF using Al^{3+} and different FA concentrations after 20 h light irradiation and in the dark. The dominant species $[Al(H_2O)_6]^{3+}$ and hydrolysed species $[Al(OH)(aq)]^{2+}$ decreased significantly as FA concentration increased and disappeared when FA concentration was over 12 g L^{-1}. This result is in line with the experimental results in Fig. 1c that show the photocatalytic activity decreasing with

the increase of FA concentration at high FA concentrations. Due to the lack of signal in the ^{27}Al NMR at high FA concentration, we infer that an insoluble aluminium fulvate coordination polymer has formed. The glucose molecules cannot contact the catalytically active Al^{3+} sites to be activated for the reaction. On the other hand, at low FA concentration, more glucose molecules can coordinate to the Al^{3+} sites (have a chance to be activated), but the light absorption is poor due to the incomplete formation of the aluminium fulvate complex. This suggests that prolonged reaction time can lead to polymerisation and removal of aluminium species from solution by precipitation.

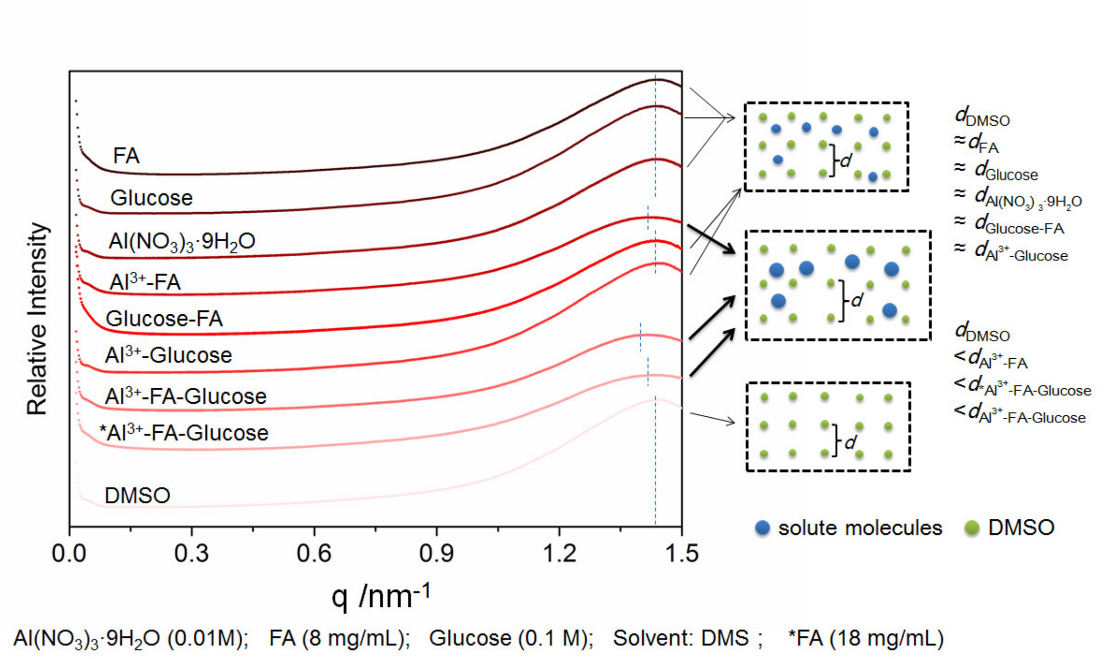

Supplementary Fig. 5. SAXS diffractogram. The bottom curve represents the results of pure DMSO solvent. It indicates that there are some periodic structures in DMSO solvent, such as aggregates formed by the interaction of several DMSO molecules (H-bonding). The abscissa represents the spacing of these aggregates in DMSO, and the smaller the coordinate value is, the larger the distance between the aggregates is.

Supplementary Fig. 5 show that for the solution of Al^{3+} and FA in DMSO, the horizontal coordinate shift to lower q indicates that Al^{3+} and FA may form large complexes through coordination, and these complexes are dispersed in DMSO aggregates, resulting in the increase of spacing between DMSO aggregates. When glucose is added to the solution of Al^{3+} and FA (8 g L^{-1}) in DMSO, the shift becomes

more obvious. We suggest that glucose, Al^{3+} and FA interact at the same time to form a larger complex, which further increases the distance between DMSO aggregate. When the FA concentration in the Al^{3+}-FA-glucose solution is increased to 18 g L^{-1}, the size of the complex formed becomes smaller than that in the solution with FA concentration of 8 g L^{-1}. This may be due to the precipitation of Al^{3+}-FA aggregates from the solution. Therefore, the phenomenon of "propping up" between DMSO aggregates is not obvious. This phenomenon seems to be related to the decrease of catalytic activity when FA increases to 18 g L^{-1}.

Section 5: The experiment under sunlight

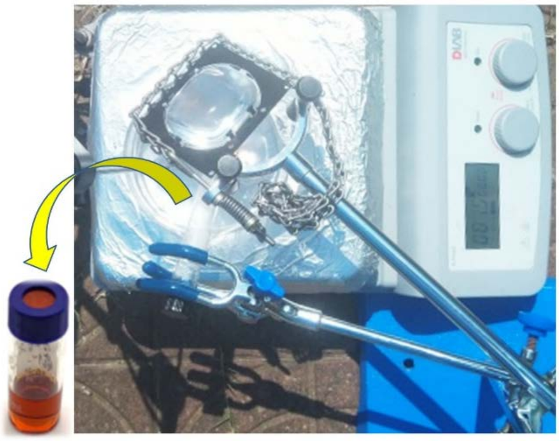

Supplementary Fig. 6. The experiment was conducted from 9:30 a.m. to 3:30 p.m. continually for 4 days. The GC vial shows the colour of the product. Reaction conditions: 0.01 M of $Al(NO_3)_3{\cdot}9H_2O$, 8 mg of FA, 0.1 M D-glucose/DMSO, ~0.29 W cm^{-2} of light intensity, 80 ^{o}C of reaction temperature, 24 h of reaction time, 1 atm of an argon atmosphere.

Section 6: Photoluminescence emission spectra of the catalytic system and its separate components

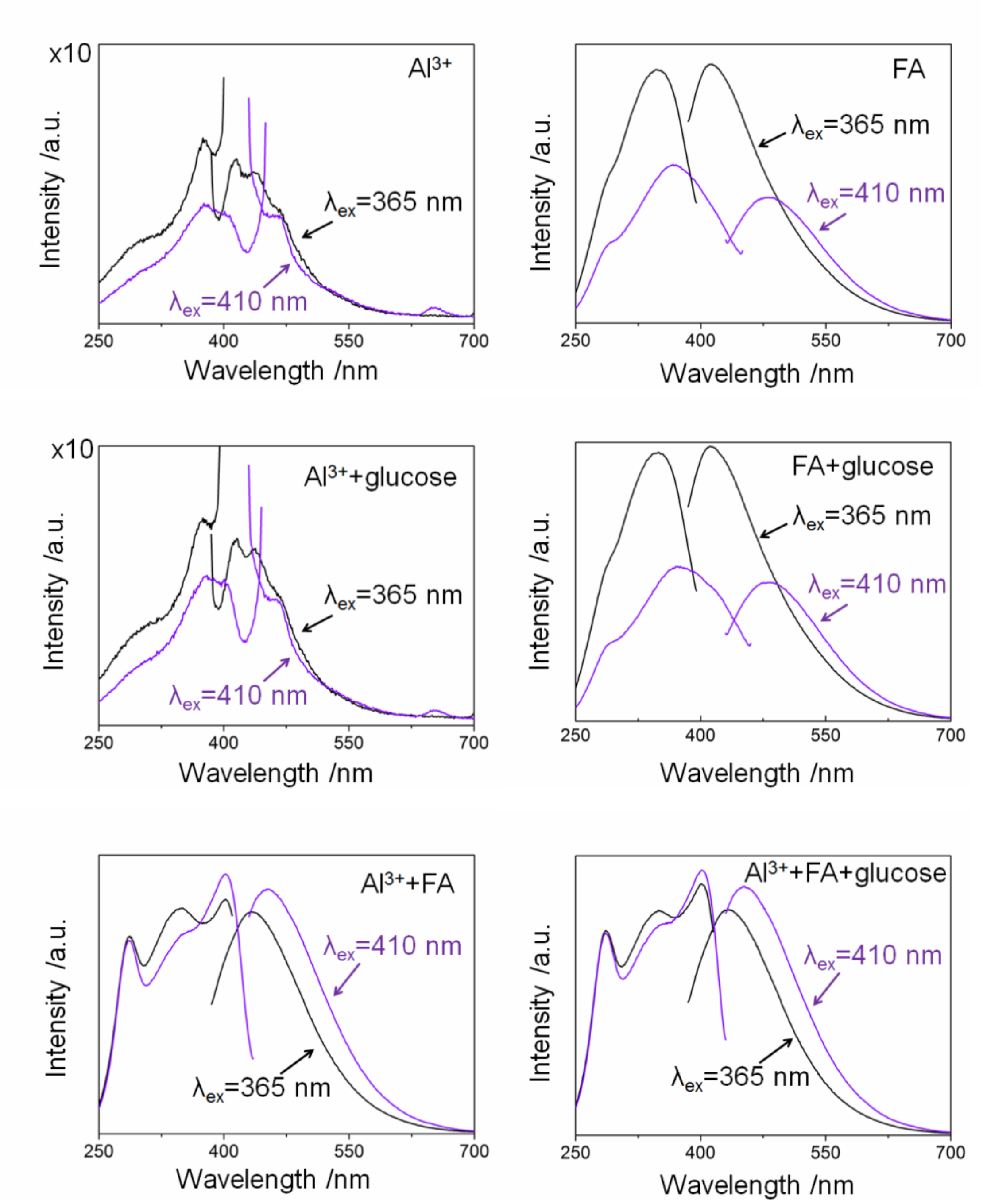

Supplementary Fig. 7. Photoluminescence excitation and emission spectra of the samples related to the FA-Al^{3+} catalytic system. The mixture was dissolved in 1 mL of DMSO and filled with argon, then stirred at 50 °C for 30 min in the dark. The mixture was diluted with DMSO by 100 times before measurement.

Further investigation of the nature of the photo-induced transition was conducted by photoluminescence spectroscopy (Supplementary Fig. 7). Thermally equilibrated DMSO solutions of both aluminium(III) nitrate and FA displayed luminescence properties upon excitation at 365 nm and 410 nm, with no further notable changes observed upon adding glucose. Of keynote in this series of experiments is the significant enhancement of emission upon excitation at 410 nm in the Al^{3+}-FA and Al^{3+}-FA-glucose systems compared to their metal-free equivalents, where emission is much weaker when excited at 410 nm compared to 365 nm. This is in line with the new feature in the absorption spectrum at 410 nm discussed in Figs. 1b-1d. We tentatively assign this feature to a charge transfer (CT) transition, as observed in the well-known [tris(8-hydroxyquinolinato)aluminium(III)] (Alq_3) and other similar complexes[9-11]. This is most likely a ligand-to-metal charge transfer (LMCT). Inspection of the excitation spectra for the Al^{3+}-FA containing samples shows a series of three broad peaks with maxima at 290, 350, and 405 nm, which likely correspond to these transitions for each of a selection of major components of FA. The most intense of these is the absorption centred at 405 nm. The broad emission peak is centred on 433 nm (λ_{ex}=365 nm) or 451 nm (λ_{ex}=410 nm), a shift from 412 and 479 nm respectively in free FA solutions.

The intensity of the emission band in the photoluminescence spectra is proportional to the with light of wavelength λ_{ex}. Given that the intensity of the exciting light with different wavelengths was identical, the peak intensity of the emission indicates the efficiency of the photons with a wavelength λ_{ex} exciting the complexes to high energy states. Under the light irradiation at 365 and 410 nm wavelengths, 43% and 55% of HMF yields were achieved, respectively. Hence, the strong correlation between yields and the excitation efficiency suggests that the catalytic reaction is caused by photoexcitation of the complexes to excited electronic states, the LMCT effect. Activity is still observed at wavelengths over 500 nm. The complex mixture of compounds in FA is likely to contain potentially coordinating conjugated species that absorb across the entirety of the spectrum, so this broad

activity is not unexpected quantity of excited electronic states of the Al^{3+}-FA-glucose complex upon irradiation

Section 7: UV-vis spectra of FA-polyphenol-glucose complexes after irradiation under different wavelengths

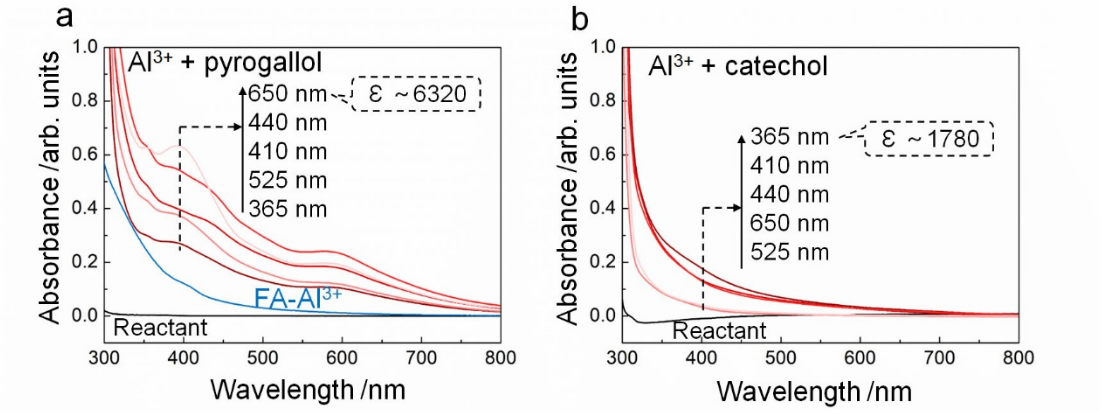

Supplementary Fig. 8. UV-vis spectra of FA-pyrogallol-glucose (a) and FA-catechol-glucose (b) complexes after irradiation under different wavelengths. UV-vis absorption spectra of the FA-Al^{3+} catalyst (blue curves) and pyrogallol/catechol-Al^{3+} catalysts before irradiation (black curves) are presented for comparison. The highest absorption intensity at 410 nm wavelength for the system of Al^{3+} and pyrogallol is observed after irradiation of 650 nm, while it is 365 nm for the system of Al^{3+} and catechol. Reaction conditions: 0.01 M $Al(NO_3)_3{\cdot}9H_2O$, 8 mg of additives, 0.1 M D-glucose/DMSO; the reactions proceed under five light sources with a light intensity of 0.2 W cm^{-2} at 70 °C for 20 h in an argon atmosphere of 1 bar. The obtained mixture was diluted 100 times with DMSO prior to spectroscopic measurement.

Post-irradiation, the UV-vis spectrum of the polyphenol solution with aluminium nitrate and D-glucose reveals an intense absorption below 400 nm, which we ascribe to the catalytically active aluminium catecholate complex. The high intensity of this peak is in good agreement with that expected of an LMCT transition in an aluminium(III) complex[12]. While we cannot accurately determine ε as the identity and concentration of the active species have not been determined, the use of the total Al^{3+} concentration affords ε of 1780 and 6320 L mol^{-1} cm^{-1} at 400 nm for catechol and pyrogallol, respectively. Given that the concentration of active species is likely lower due to incomplete coordination of aluminium to catechol, this calculated absorption coefficient is a lower bound on the 'true' ε of the complex.

Section 8: NMR studies on the interaction of FA with Al^{3+}

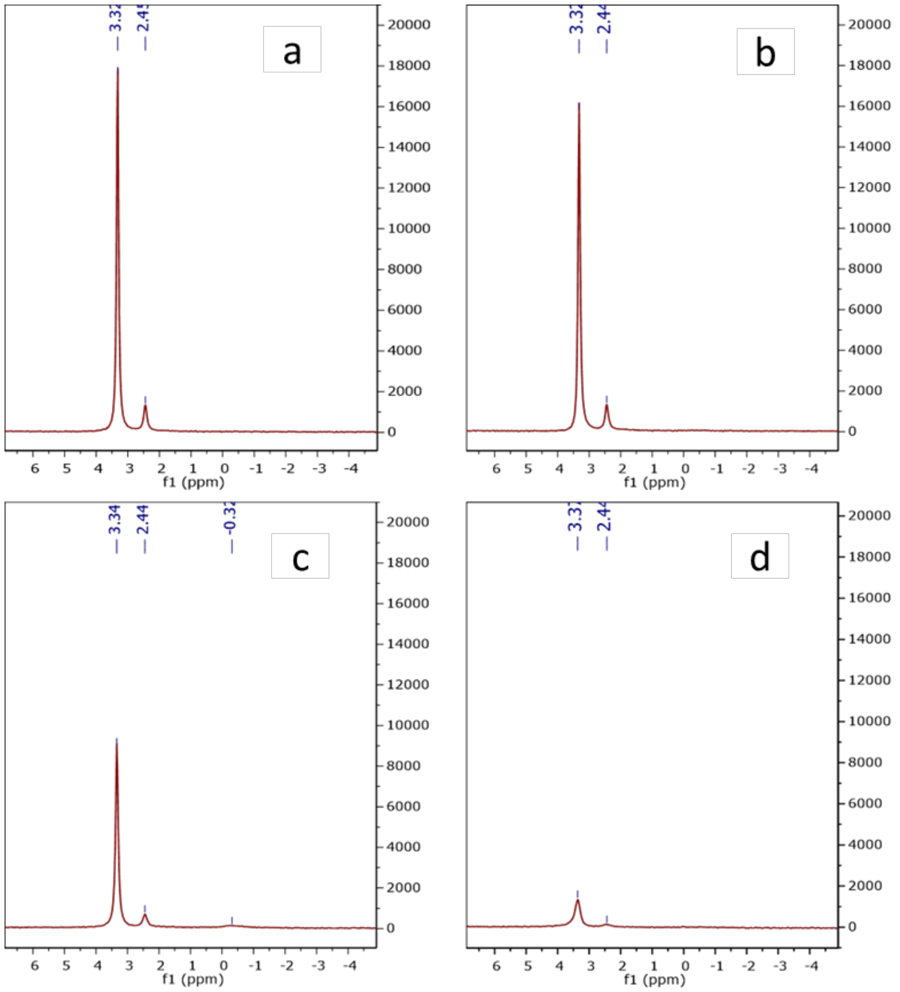

Supplementary Fig. 9. ^{27}Al NMR spectra of A) 0.013 M $Al(NO_3)_3 \cdot 9H_2O$ and 0.1 M glucose in DMSO irradiated with light (1.0 W cm^{-2}) at 60°C for 4 h, B) above solution irradiated under light for 8 h, C) 4.5 g L^{-1} FA and 0.013 M $Al(NO_3)_3 \cdot 9H_2O$ and 0.1 M glucose in DMSO, irradiated under light (1.0 W cm^{-2}) at 60 °C for 4 h, D) 8 h.

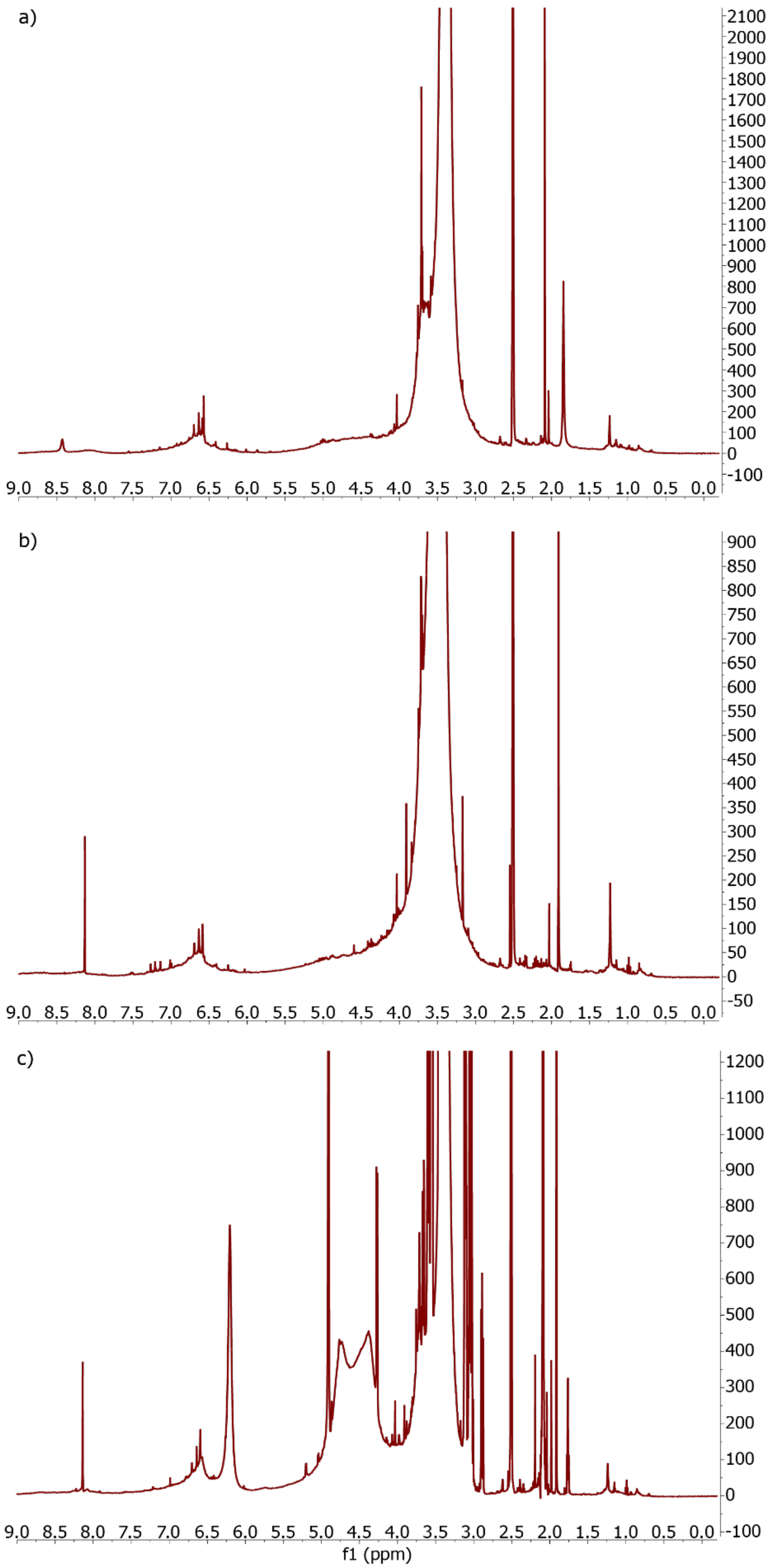

Supplementary Fig. 10. ^{1}H NMR spectra of a) FA (8 g L^{-1}), b) FA (8 g L^{-1}) and $Al(NO_3)_3 \cdot 9H_2O$ (0.01 M), c) FA (8 g L^{-1}), $Al(NO_3)_3 \cdot 9H_2O$ (0.01 M) and glucose (0.1 M) in DMSO-D_6 (0.75 mL). Aluminium-containing solutions were stirred for 16 h in the dark to allow for equilibration.

A series of DMSO-d_6 solutions containing 0.01 M aluminium nitrate and either 8 g L^{-1} FA, 0.1 M D-glucose or both FA and D-glucose were prepared and allowed to equilibrate by stirring in the dark at room temperature for 16 h. Comparison of NMR spectra after a further 24 h confirmed that these samples had reached equilibrium. Both 1D ^{1}H and ^{27}Al NMR spectra were then examined for these solutions. Due to the complexity of the ^{1}H NMR spectrum of FA, the ^{27}Al NMR spectra were initially more instructive. Inspection and partial deconvolution of the ^{27}Al NMR spectrum (Supplementary Fig. 8c) revealed the presence of the previously observed aquated Al^{3+} cations at 3.3 and 2.4 ppm in addition to at least five other aluminium species, all octahedrally coordinated. Given the low concentrations involved, as well as the relatively broad peak shape inherent to ^{27}Al NMR, it is likely that there are further signals that cannot be reliably deconvoluted. This is not an unexpected finding, given the complex nature of FA mixtures, which can contain over 3000 unique molecular components.[13]

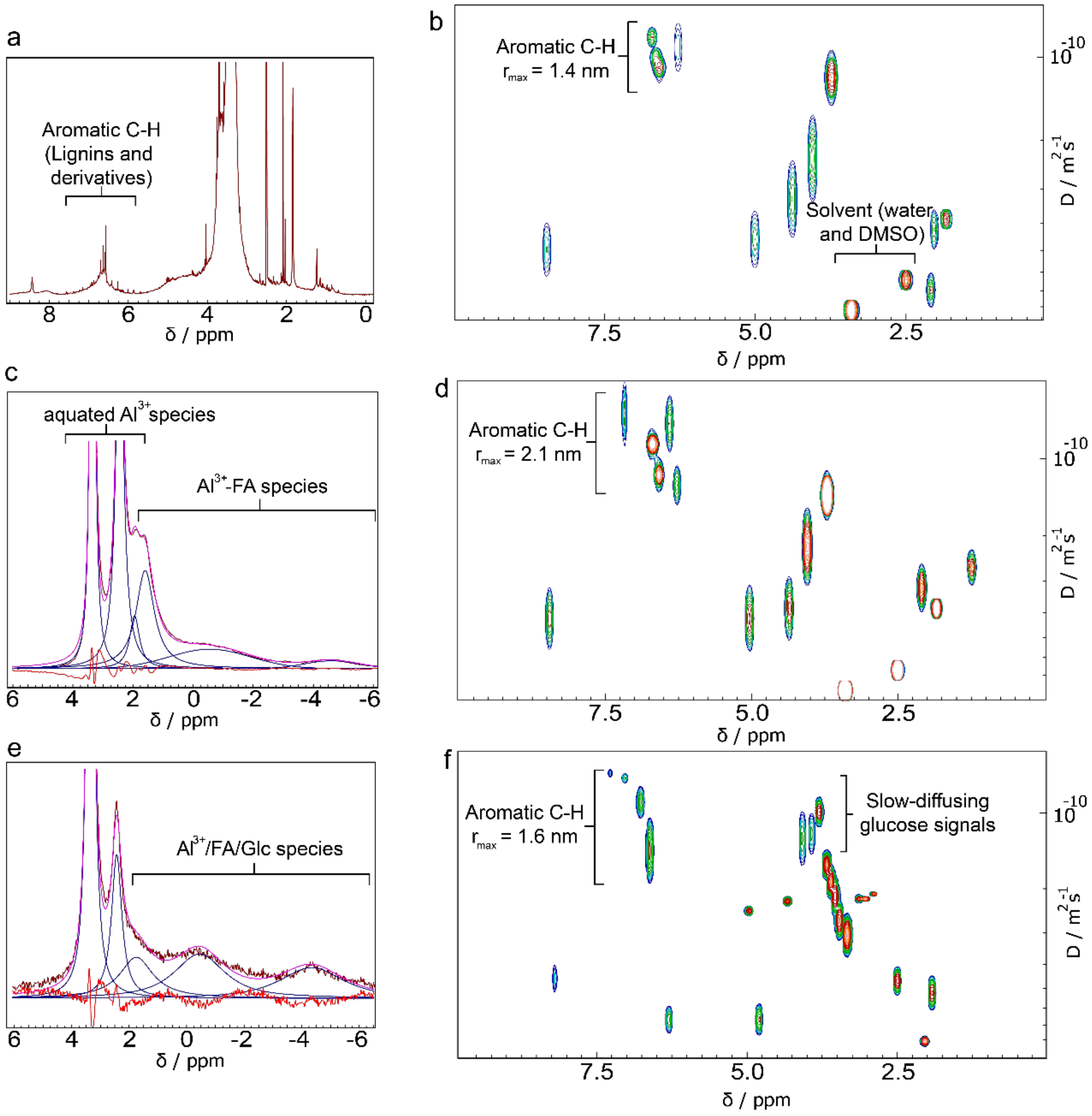

Supplementary Fig. 11. a) ^{1}H NMR and b) DOSY NMR of FA (8 g L^{-1} in D_6-DMSO); c) ^{27}Al and d) DOSY NMR of FA-Al^{3+} solution (8 g L^{-1} FA and 0.01 M $Al(NO_3)_3{\cdot}9H_2O$ in D_6-DMSO); e) ^{27}Al and f) DOSY NMR of FA-Al^{3+}-glucose solution (8 g L^{-1} FA, 0.01 M $Al(NO_3)_3{\cdot}9H_2O$ and 0.1 M glucose in D_6-DMSO). Solvodynamic radii calculated using Stokes-Einstein relation. The scheme at bottom shows schematically the potential coordination spheres of octahedrally coordinated Al^{3+} cations.

Further analysis of the nature of FA coordination to Al^{3+} was obtained through 2D DOSY NMR spectroscopy (Figs. S11b, S11d, S11f) and ^{1}H NMR spectroscopy (Supplementary Fig. 10). DOSY of FA shows a broad range of diffusion coefficients, corresponding to solvodynamic radii ranging from 0.19 to 1.43 nm as determined by the Stokes-Einstein relation[14,15]. This size variation agrees with the broad range of molecular weights observed in the gel permeation chromatography (GPC) analysis

(Supplementary Fig. 13), as FA components range from small organic molecules to macromolecular species such as lignins. Upon adding Al^{3+} (Supplementary Fig. 11d), the observed solvodynamic radii increase, with the largest species measuring 2.1 nm in radius, consistent with the formation of Al^{3+} complexes with ligands of the FA components. This size increase is most notable in the aromatic region of the ^{1}H NMR spectrum, which is expected due to the coordination of lignin-derived polyphenolates. The addition of glucose to this mixture (Supplementary Fig. 11f) led to smaller complexes (maximum observed radius of 1.57 nm). We propose that this is due to the smaller glucose ligands replacing some of the polynucleating FA ligands. This is supported by the observed increase in diffusion coefficient of some glucose-related signals in the DOSY NMR. Full resolution of this is unfortunately hindered by the high degree of overlap with uncoordinated glucose and other FA-derived signals. Small-angle X-ray Scattering (SAXS) analysis (Supplementary Fig. 5) also supports the formation of complexes in Al^{3+}-FA and Al^{3+}-FA-glucose mixtures.

The nature of FA interaction with aluminium(III) was also examined by NMR studies. In the ^{27}Al NMR study, the octahedral coordination remained when FA was added in the Al^{3+}/DMSO solution to a concentration of 8 g L^{-1} while a number of new Al^{3+} species with different ligands were observed (Supplementary Fig. 11c). When 0.1M glucose is included in a solution of FA (8 g L^{-1}) and Al^{3+} (0.01M) in DMSO, at least two additional octahedrally coordinated aluminium species can be observed in addition to the ‘uncoordinated’ aquated species seen in other samples (Supplementary Fig. 11e). These species do not appear to match those observed in a similar solution of solely aluminium nitrate and glucose (Supplementary Figs. 4 and 9a) and thus likely correspond to Al^{3+}-FA-glucose complexes of potential interest. This suggests that glucose molecules most likely replace some weakly coordinated ligands from FA, coordinating with Al^{3+} ions.

Section 9: Calculated frontier molecular orbitals for aluminium(III)-pyrogallol-glucose complexes

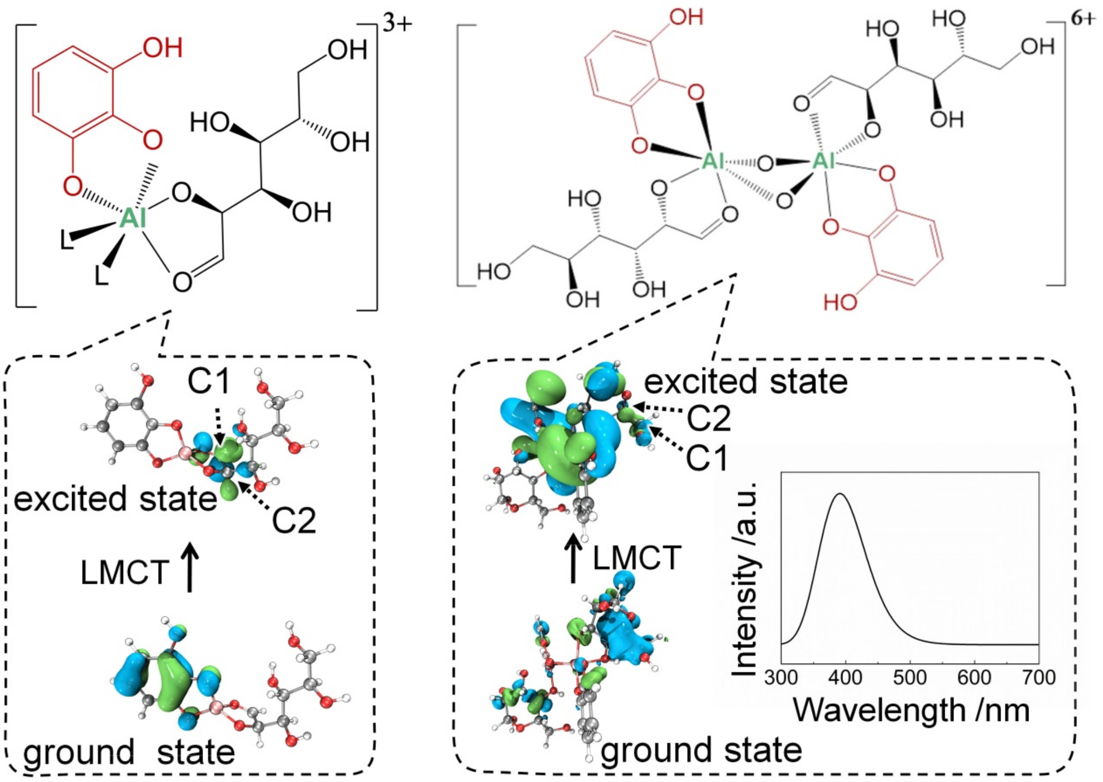

Supplementary Fig. 12. The calculated frontier molecular orbitals for aluminium(III)-pyrogallol-glucose complexes. Coordinated ligand L is bidentate bridging D-glucose, weakly coordinated water, DMSO solvent or non-catalytic, weakly bound FA components. The inset shows the UV-vis absorption electronic spectrum of the binuclear Al-pyrogallol-glucose complex simulated by the Time-Dependent Density Functional Theory (TD-DFT) procedure. Calculations were conducted using DFT (B3LYP/6-31G+(d)) within Gaussian 16.

Section 10: GPC analysis of FA

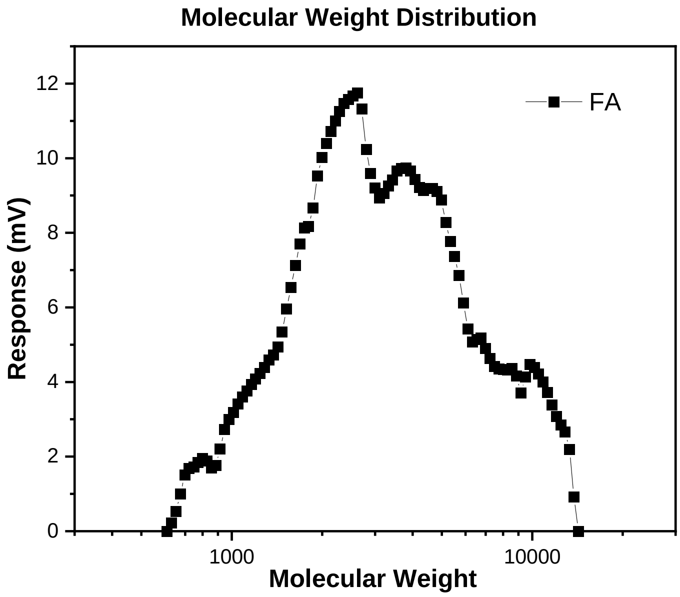

Supplementary Fig. 13. GPC trace of FA used in this study. Molecular weights were determined by comparison with Pullulan standards. Mn = 2537, Mw = 3952, PDI = 1.56.

Gel permeation chromatography (GPC) analysis (Supplementary Fig. 13) shows that the FA used in this study consists of a range of compounds from small molecules through to larger oligomeric and polymeric lignin-derived species. From this data, number-average and weight-average molecular weights can be determined and used to calculate representative molar absorption coefficients (ε). However, coefficients calculated by this method do not necessarily reflect the true concentration of the actively absorbing aluminium complex within the solution and serve simply as an ‘apparent’ ε based on the total quantity of fulvic acid present. The active aluminium(III) complex is likely to present in a much lower concentration, and as such, the ‘true’ ε for this charge transfer transition will be much higher. This is further reinforced by the calculation of ε based on total aluminium(III) concentration (96.15 M^{-1} cm^{-1}), which is incongruent with the likely charge-transfer nature of the transition.

Section 11: Solvent effect

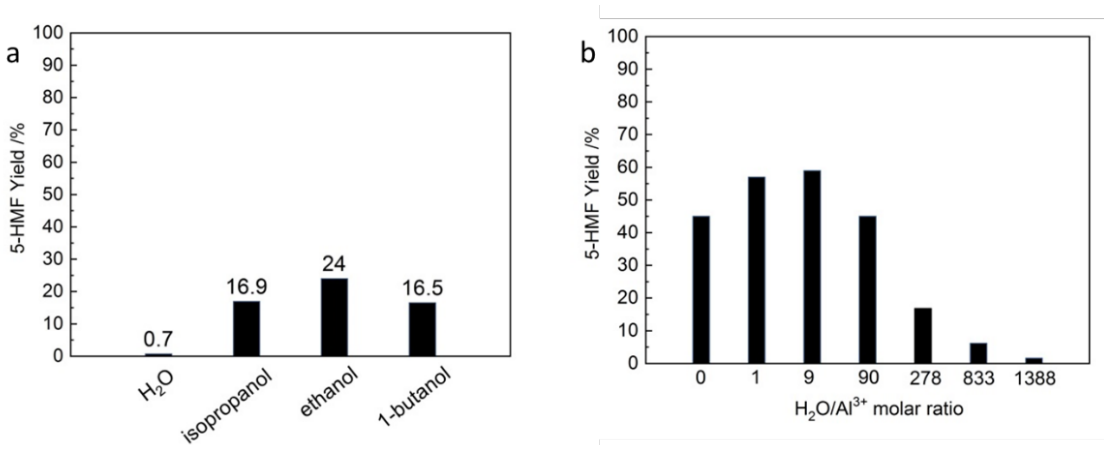

Supplementary Fig. 14. The result of glucose conversion in cosolvent system and the impact of water content on HMF yield under light irradiation. Reaction conditions: **a,** 0.01 M of aluminium salt, 8.0 g L^{-1} fulvic acid, 0.1 M D-glucose, 1 mL of DMSO and 1 mL of the other medium (water, isopropanol, ethanol or 1-butanol) were used as the cosolvent, 1.2 W cm^{-2} of light intensity (400-800 nm wavelength), 80 ^{o}C, 20 h, 1 atm argon atmosphere; **b**, 2 mL of DMSO or H_2O-DMSO cosolvent.

The glucose conversion in the cosolvent system was also conducted, as seen in the Supplementary Fig. 14a. The addition of other organic solvents like isopropanol, ethanol and 1-butanol all reduced the HMF yield obviously, and nearly no HMF can be obtained after addition of water. For solvent effects, DMSO could interact with proton, forming more active catalytic species to enhance the dehydration of fructose to HMF[16]. DMSO solvent also could elevate the energy of the lowest unoccupied molecular orbital of HMF, thus making HMF less susceptible to nucleophilic attack, resulting in formation of HMF as the primary product[17].

The addition of a small amount of water in the reaction mixture resulted in the optimised yield of HMF (Supplementary Fig. 14b), higher water content in the reaction system reduced the HMF yield that may due to the lower stability of the HMF in water[18].

Section 12: Carbon balance determination

Supplementary Table 2. Mass balance of the transformation of D-glucose to HMF under visible-light irradiation and in the dark

Entry	Condition	Conversion (%)	Fructose (%)	HMF (%)	Carbon balance (%)
1	Light	83.5	0	65	81.5
2	Dark	38	13	11	86

Reaction conditions: a 0.01 M of $Al(NO_3)_3 \cdot 9H_2O$, 8.0 g L^{-1} fulvic acid, 0.1 M D-glucose/DMSO, 1.2 W cm^{-2} of light intensity (400-800 nm wavelength), 80 °C, 20 h, 1 atm argon atmosphere. Dark reactions were conducted at 80 °C.

The mass balance was determined and shown in Supplementary Table 2. Based on conversion of substrates and products yield, the carbon balance for the light reaction is calculated to be 81.5%, while that for dark reaction is 86%. This suggests that there are some by-products, such as humin formation during the D-glucose conversion.

References

1 Lemoine, P., Bekaert, A., Brion, J. & Viossat, B. Crystal structure of hexakis (μ_2-acetato)-tris (acetonitrile-κN)-μ_3-oxotrialuminum (III) tetrachloroaluminate, $[Al_3(C_2H_3O_2)_6(C_2H_3N)_3O][AlCl_4]$. *Zeits. fur Krist.* **221**, 309-310 (2006).

2 Piskunov, A. V. *et al.* Quinone complexes of aluminum: synthesis and structures. *Russ. J. Coord. Chem.* **36**, 161-169 (2010).

3 Choudhary, V. *et al.* Insights into the interplay of Lewis and Brønsted acid catalysts in glucose and fructose conversion to 5-(hydroxymethyl) furfural and levulinic acid in aqueous media. *J. Am. Chem. Soc.* **135**, 3997-4006 (2013).

4 Tang, J., Guo, X., Zhu, L. & Hu, C. Mechanistic study of glucose-to-fructose isomerization in water catalyzed by $[Al(OH)_2(aq)]^+$. *ACS Catal.* **5**, 5097-5103 (2015).

5 De, S., Dutta, S. & Saha, B. Microwave assisted conversion of carbohydrates and biopolymers to 5-hydroxymethylfurfural with aluminium chloride catalyst in water. *Green Chem.* **13**, 2859-2868 (2011).

6 Pagan-Torres, Y. J. *et al.* Production of 5-hydroxymethylfurfural from glucose using a combination of Lewis and Brønsted acid catalysts in water in a biphasic reactor with an alkylphenol solvent. *ACS Catal.* **2**, 930-934 (2012).

7 Gallo, J. M. R., Alonso, D. M., Mellmer, M. A. & Dumesic, J. A. Production and upgrading of 5-hydroxymethylfurfural using heterogeneous catalysts and biomass-derived solvents. *Green Chem.* **15**, 85-90 (2013).

8 Roman-Leshkov, Y., Chheda, J. N. & Dumesic, J. A. Phase modifiers promote efficient production of hydroxymethylfurfural from fructose. *Science* **312**, 1933-1937 (2006).

9 Hoshi, T. *et al.* Electronic absorption and emission spectra of Alq_3 in solution with special attention to a delayed fluorescence. *J. Lumin.* **128**, 1353-1358 (2008).

10 Kikkeri, R., Hossain, L. H. & Seeberger, P. H. Supramolecular one-pot approach to fluorescent glycodendrimers. *Chem. Commun.*, 2127-2129 (2008).

11 Baleizão, C. *et al.* Photochemistry of chiral pentacoordinated Al salen complexes. Chiral recognition in the quenching of photogenerated tetracoordinated Al salen transient by alkenes. *Photochem. Photobiol. Sci.* **2**, 386-392 (2003).

12 Gualandi, A. *et al.* Aluminum(III) salen complexes as active photoredox catalysts. *Eur. J. Org. Chem.* **2020**, 1486-1490 (2020).

13 Han, R. *et al.* Molecular-scale investigation of soil fulvic acid and water-extractable organic matter by high-resolution mass spectrometry and 1H NMR spectroscopy. *Environ. Chem.* **16**, 92-100 (2019).

14 Einstein, A. The motion of elements suspended in static liquids as claimed in the molecular kinetic theory of heat. *Ann. Phys. (Berlin)* **17**, 549-560 (1905).

15 Sutherland, W. LXXV. A dynamical theory of diffusion for non-electrolytes and the molecular mass of albumin. *Philos. Mag.* **9**, 781-785 (1905).

16 Ren, L.-K. *et al.* Performance of dimethyl sulfoxide and Brønsted acid catalysts in fructose conversion to 5-hydroxymethylfurfural. *ACS Catal.* **7**, 2199-2212 (2017).

17 Tsilomelekis, G., Josephson, T. R., Nikolakis, V. & Caratzoulas, S. Origin of 5-hydroxymethylfurfural stability in water/dimethyl sulfoxide mixtures. *ChemSusChem* **7**, 117-126 (2014).

18 Despax, S. *et al.* Fast and efficient DMSO-mediated dehydration of carbohydrates into 5-hydroxymethylfurfural. *Catal. Commun.* **51**, 5-9 (2014).
